# Regulation of inflammatory signaling by the ST6Gal-I sialyltransferase

**DOI:** 10.1371/journal.pone.0241850

**Published:** 2020-11-09

**Authors:** Andrew T. Holdbrooks, Katherine E. Ankenbauer, Jihye Hwang, Susan L. Bellis

**Affiliations:** Department of Cell, Developmental and Integrative Biology, University of Alabama at Birmingham, Birmingham, AL, United States of America; INSERM, FRANCE

## Abstract

The ST6Gal-I sialyltransferase, an enzyme that adds α2-6-linked sialic acids to *N*-glycosylated proteins, regulates multiple immunological processes. However, the contribution of receptor sialylation to inflammatory signaling has been under-investigated. In the current study, we uncovered a role for ST6Gal-I in promoting sustained signaling through two prominent inflammatory pathways, NFκB and JAK/STAT. Using the U937 monocytic cell model, we determined that knockdown (KD) of ST6Gal-I expression had no effect on the rapid activation of NFκB by TNF (≤ 30 min), whereas long-term TNF-induced NFκB activation (2–6 hr) was diminished in ST6Gal-I-KD cells. These data align with prior work in epithelial cells showing that α2–6 sialylation of TNFR1 prolongs TNF-dependent NFκB activation. Similar to TNF, long-term, but not short-term, LPS-induced activation of NFκB was suppressed by ST6Gal-I KD. ST6Gal-I KD cells also exhibited reduced long-term IRF3 and STAT3 activation by LPS. Given that ST6Gal-I activity modulated LPS-dependent signaling, we conducted pull-down assays using SNA (a lectin specific for α2–6 sialic acids) to show that the LPS receptor, TLR4, is a substrate for sialylation by ST6Gal-I. We next assessed signaling by IFNγ, IL-6 and GM-CSF, and found that ST6Gal-I-KD had a limited effect on STAT activation induced by these cytokines. To corroborate these findings, signaling was monitored in bone marrow derived macrophages (BMDMs) from mice with myeloid-specific deletion of ST6Gal-I (LysMCre/ST6Gal-I^fl/fl^). In agreement with data from U937 cells, BMDMs with ST6Gal-I knockout displayed reduced long-term activation of NFκB by both TNF and LPS, and diminished long-term LPS-dependent STAT3 activation. However, STAT activation induced by IFNγ, IL-6 and GM-CSF was comparable in wild-type and ST6Gal-I knockout BMDMs. These results implicate ST6Gal-I-mediated receptor sialylation in prolonging the activity of select signaling cascades including TNF/NFκB, LPS/NFκB, and LPS/STAT3, providing new insights into ST6Gal-I’s role in modulating the inflammatory phenotype of monocytic cells.

## Introduction

Sialyltransferases play a seminal role in a variety of immunological processes through their modulation of surface receptors. For example, ST6Gal-I, a Golgi sialyltransferase that adds α2–6 linked sialic acids to *N*-glycosylated proteins, has been shown to be vital for granulopoiesis [1, 2], thymopoiesis [3], B cell proliferation [4] and antibody production [4, 5]. Moreover, genome-wide association studies (GWAS) have revealed that single nucleotide polymorphisms of ST6Gal-I are linked to multiple immune-related disorders, including multiple sclerosis [6], coronary artery disease [7], type 2 diabetes [8, 9] and IgA nephropathy [10]. While many studies have examined the role of ST6Gal-I-mediated sialylation in modulating immune endpoints, limited attention has been paid to the mechanisms by which sialylation affects inflammatory signal transduction.

One of the most highly studied inflammatory signaling pathways is the NFκB axis. Pivotal to controlling multiple facets of innate and adaptive immunity, the NFκB transcription factor induces the expression of numerous pro-inflammatory genes [11]. Dysregulation of the NFκB pathway fosters a myriad of inflammatory diseases [11, 12]. NFκB signaling can be initiated by a variety of stimuli, however, two of the primary activators of NFκB are tumor necrosis factor (TNF) and lipopolysaccharide (LPS), a constituent of the outer membrane of Gram-negative bacteria. TNF and LPS both play central roles in regulating the activation of immune cells such as monocytes and macrophages. TNF stimulation of tumor necrosis factor receptor 1 (TNFR1), the receptor responsible for most TNF signaling events [13, 14], leads to the immediate recruitment of Complex I proteins, including TRADD, TRAF2, RIP1 and cIAP1/2, and subsequent activation of NFκB [15, 16]. However, following these events, TNFR1 internalizes into the endosome, where Complex I is replaced by Complex II proteins (FADD, caspase 8), which are responsible for initiating caspase-mediated apoptosis [15, 16]. In the LPS signaling pathway [17], LPS initially interacts with shuttle proteins that subsequently facilitate LPS binding to the TLR4/MD-2 receptor complex. TLR4 then oligomerizes, prompting the recruitment of adaptor proteins such as MyD88. During early TLR4 signaling, MyD88 is the key adaptor protein responsible for the activation of NFκB. Later on, TLR4 internalizes, leading to the recruitment of TRIF, which promotes activation of both NFκB and IRF3. In addition, LPS stimulation of TLR4 can indirectly activate JAK/STAT signaling [18, 19]. Similar to NFκB, the JAK/STAT pathway promotes expression of a wide array of inflammatory genes, and is also the principal system through which numerous cytokines and growth factors signal [20].

Prior studies from our group have shown that signaling by TNFR1 is modulated by ST6Gal-I-mediated α2–6 sialylation. In both epithelial cancer cells [21] and U937 monocytic cells [22], TNF-induced apoptosis is blocked by α2–6 sialylation of TNFR1. The mechanism appears to be due to a sialylation-dependent impairment in TNFR1 internalization [21]. This consequently diverts downstream signaling toward sustained NFkB activation [21], given that TNFR1 internalization is required for apoptosis [23–25]. These results are consistent with other studies in which inhibitors of TNFR1 internalization prevented caspase-mediated apoptosis, while simultaneously prolonging the activation of NFκB [23–25].

As with TNFR1, the sialylation status of TLR4 is reported to affect its activation of NFκB [26]. However, in prior studies, the functional effects of TLR4 sialylation were inferred through the use of sialidases, enzymes that cleave sialic acids from glycoconjugates. Sialidases are not specific for the α2–6 sialic acid linkage, and therefore the explicit contribution of ST6Gal-I in regulating the LPS/TLR4 axis has yet to be investigated. Additionally, the effect of sialylation on JAK/STAT signaling remains poorly understood. In the present investigation, we define novel roles for ST6Gal-I in regulating TNF and LPS-induced inflammatory signaling in cells of the monocytic lineage. Using U937 monocytic cells with ST6Gal-I knockdown (KD), or bone marrow-derived macrophages (BMDMs) from mice with ST6Gal-I knockout, we show that loss of ST6Gal-I expression leads to a decrease in sustained activation of NFκB induced by LPS and TNF. Furthermore, sustained activation of IRF3 and STAT3 by LPS is also attenuated in ST6Gal-I knockdown/knockout cells. These data provide new insights into the mechanisms by which α2–6 sialylation regulates immune cell function.

## Materials and methods

### U937 cell lines

U1 cells were purchased from ATCC and maintained in Dulbecco’s modified Eagle’s medium containing 10% fetal bovine serum (FBS) and 1% antibiotic/antimycotic supplements (A/A, GE Healthcare Hyclone). A stable, polyclonal ST6Gal-I KD cell line was created by transducing U937 cells with lentivirus encoding a shRNA against ST6Gal-I (Sigma, TN00000035432, sequence CCGGCGTGTGCTACTACTACCAGAACTCGAGTTCTGGTAGTAGTAGCACACGTTTTTG), followed by selection with 1 μg/ml of puromycin (Sigma). As a control, cells were stably transduced with an empty lentiviral vector. Puromycin was removed from the medium at least 2 days prior to all experiments.

### Mice with conditional deletion of *St6gal1*

C57BL/6 mice homozygous for the floxed *St6gal1* gene (B6.129-St6gal1^tm2Jxm^/J) were purchased from The Jackson Laboratory. These mice were crossed to LysM-Cre mice (The Jackson Laboratory, B6.129P2-Lyz2^tm1(cre)Ifo^/J) to generate myeloid-specific deletion of *St6gal1*. Control animals consisted of floxed *St6gal1* mice with no Cre-recombinase expression. All animal experiments were conducted with prior approval from the University of Alabama at Birmingham (UAB) Institutional Animal Care and Use Committee.

### BMDM isolation and culture

Bone marrow cells were flushed from the femurs of 8-12-week-old mice. Following red blood cell lysis (ACK Lysing Buffer, Gibco), cells were seeded in tissue culture treated dishes and incubated for 3–4 hr. Suspension cells were then pelleted and cultured in RPMI 1640 medium containing 10% FBS, 1% A/A and 20 ng/ml of murine M-CSF (R&D Systems) for 4–6 days to obtain BMDMs.

### Cell treatments

All cell treatments were performed in medium containing 1% serum (2 hr pre-incubation in 1% serum-containing medium, followed by administration of stimulatory factors in 1% serum). To examine signaling, cells were cultured for the indicated times with 10 ng/ml of each molecule–TNF (R&D Systems), LPS (Sigma), IFNγ, IL-6 and GM-CSF (all from Peprotech).

### Immunoblotting

Cells were treated for indicated times and immediately lysed in radioimmune precipitation assay (RIPA) buffer supplemented with protease and phosphatase inhibitors (Pierce). Total protein concentration was measured by BCA (Pierce). Samples were resolved by SDS-PAGE and transferred to polyvinylidene difluoride (PVDF) membranes. Membranes were blocked with 5% nonfat dry milk in TBS buffer containing 0.1% Tween 20 (TBS-T), and then incubated with antibodies against ST6Gal-I (R&D Systems, AF5924), pNFκB–p65 (S536, Cell Signaling Technology, 3033), total NFκB–p65 (Cell Signaling Technology, 8242), p-STAT1 (Y701, Cell Signaling Technology, 7649), total STAT1 (Cell Signaling Technology, 14994), p-STAT3 (Y705, Cell Signaling Technology, 9145), total STAT3 (Cell Signaling Technology, 9139), p-STAT5 (Y694, Cell Signaling Technology, 4322), total STAT5 (Cell Signaling Technology, 94205), p-IRF3 (Cell Signaling Technology, 37829), total IRF3 (Cell Signaling Technology, 11904) and TLR4 (Novus, NB100-56566). Protein loading was verified using horseradish peroxidase (HRP)–conjugated anti-actin (Abcam, ab20272) or HRP-conjugated anti-tubulin (Abcam, ab21058). Membranes were incubated with appropriate HRP-coupled secondary antibodies (anti-rabbit and anti-mouse IgG, Cell Signaling Technology; anti-goat IgG, Santa Cruz Biotechnology), and protein was detected by ECL (Pierce), Clarity (Bio-Rad), or SuperSignal West Femto substrate (Pierce). At least two independent experiments for each cell treatment and associated immunoblots were performed.

### SNA staining

To confirm that ST6Gal-I knockdown corresponded with a decrease in α2–6 sialic acid on the cell surface, staining was performed with SNA lectin, which binds to α2–6 sialic acid. Briefly, cells were incubated with FITC-conjugated SNA (Vector, B-1305) at a 1:400 dilution for 20 minutes at 4°C. SNA staining was then quantified via flow cytometry using an LSR-II (BD Bioscience).

### SNA lectin precipitation

To verify that TLR4 is a substrate for ST6Gal-I and that α2–6 sialylation of TLR4 corresponds with ST6Gal-I manipulation, 400 μg of U937 cell lysates (harvested and quantified as previously described) were incubated with 50 μl of SNA-conjugated agarose beads (Vector, AL-1303) overnight at 4°C. α2–6–sialylated proteins bound to the beads were then precipitated by centrifugation, washed with PBS, resuspended in 1x SDS-PAGE sample buffer (Invitrogen) plus 10% 2-mercaptoethanol (Sigma) and incubated at 95°C for 5 min. Proteins were resolved by SDS-PAGE and immunoblotted for TLR4 (Novus, NB100-56566).

### Quantitative Real-Time PCR (qRT-PCR)

RNA was extracted from U937 cells treated for 6 hr with TNF or LPS using the protocol for the PureLink RNA Mini Kit (Invitrogen). Total RNA concentration was measured, and cDNA was synthesized using the M-MLV Reverse Transcriptase protocol (Promega). qPCR samples were prepared using TaqMan Fast Advanced Master Mix (Thermo). Primers for IL-6 (Hs00985639_m1), IL-8 (Hs01555410_m1), and TNF (Hs00174128_m1) were obtained from Applied Biosystems. The StepOne Plus Real-Time PCR System (Applied Biosystems) was used to determine mRNA levels. The data were analyzed using the comparative CT method to obtain relative quantitation values, which were normalized to GAPDH (Applied Biosystems, Hs02758991_gl). Values for cells treated with TNF or LPS were normalized to values for untreated cells to yield fold increases in cytokine induction. At least three independent experiments were conducted, with each independent experiment performed in triplicate.

### Enzyme-linked immunosorbent assay (ELISA)

U937 cells were treated with TNF or LPS for 6 hr and cell culture supernatants were collected. As a control, supernatants from untreated cells were also collected 6 hr after seeding. ELISA kits for IL-6 (430504, Biolegend), IL-8 (431504, Biolegend) and TNF (430204 Biolegend) were used to measure cytokine abundance in conditioned media. At least three independent experiments were conducted, with each independent experiment performed in triplicate.

## Results

### Knockdown of ST6Gal-I expression decreases sustained TNF-induced NFκB activation

**Fig 1:**
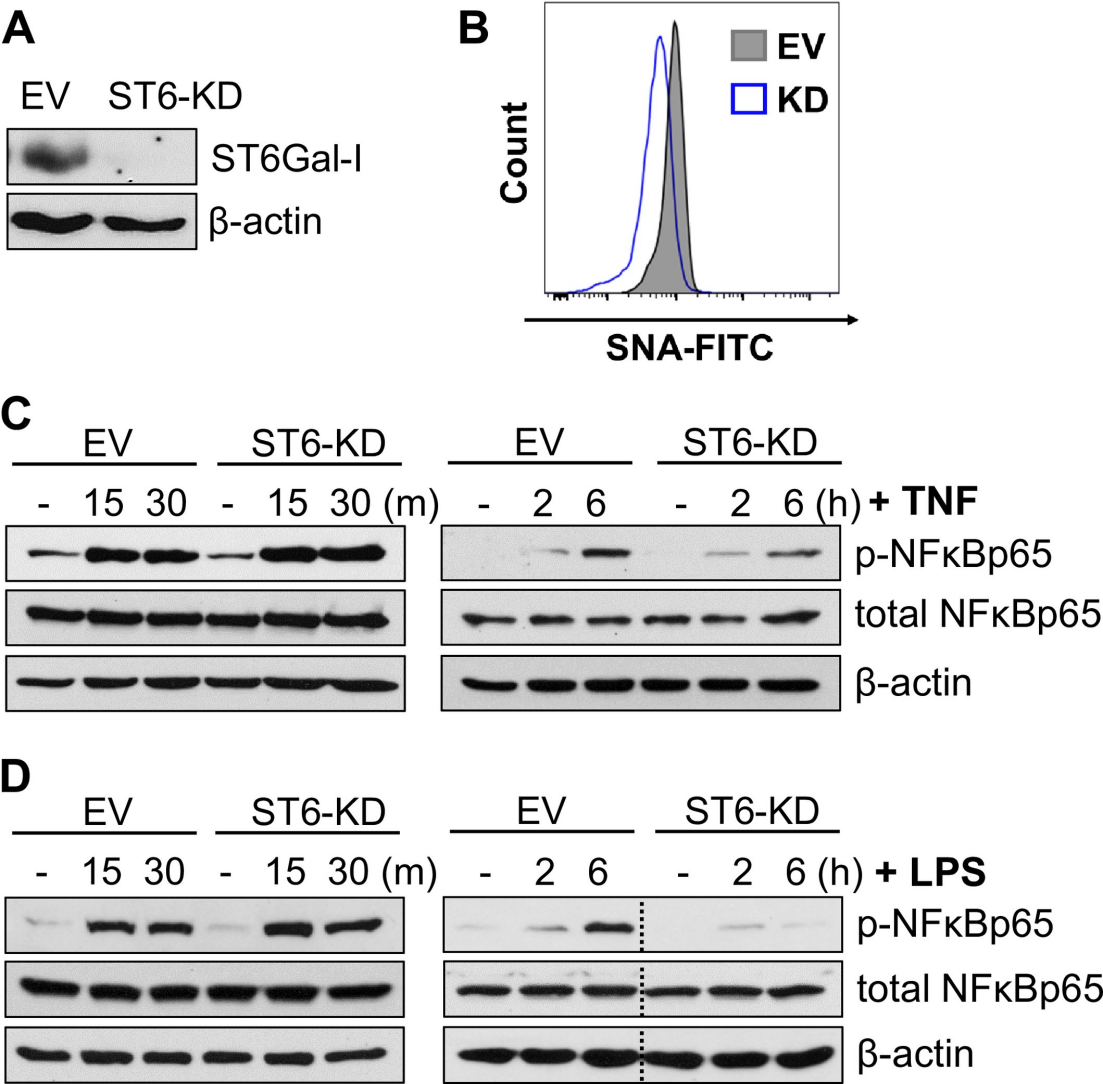
Knockdown of ST6Gal-I expression decreases sustained activation of NFκB by TNF and LPS. (A) U937 monocytic cells were stably transduced with lentivirus encoding shRNA for ST6Gal-I. Control cells were generated by transduction of an empty vector (EV) lentiviral construct. ST6Gal-I knockdown (ST6-KD) was confirmed by immunoblotting. (B) Surface sialylation levels were measured by staining cells with SNA-FITC, followed by flow cytometry. (C) U937 EV and ST6-KD cells were treated with TNF for 15–30 min (“short-term” timepoints), or 2–6 hr (“long-term” timepoints), and lysates were immunoblotted for phospho-NFκB (p-NFκBp65) or total NFκB (NFκBp65). (D) EV and ST6-KD cells were treated with LPS for short-term or long-term timepoints and lysates were immunoblotted for p-NFκBp65 or total NFκBp65. Dotted lines indicate that blots were spliced to remove irrelevant, intervening lanes (i.e., all lanes shown were on the same blot).

In prior studies using epithelial cancer cell models, we determined that α2–6 sialylation of TNFR1 had no effect on the initial activation of NFκB by TNF, but instead prolonged TNF-dependent NFκB activation [21]. To study the role of ST6Gal-I in monocyte signaling, we transduced the U937 human monocytic cell line, which expresses high endogenous levels of ST6Gal-I, with lentivirus encoding shRNA for ST6Gal-I. Stable knockdown of ST6Gal-I (ST6-KD) was confirmed by immunoblotting (Fig 1A, EV = empty vector control). To verify that knockdown of ST6 led to a concomitant decrease in cell surface sialylation, EV and ST6-KD cells were stained using SNA, a lectin specific to α2–6 sialic acid (Fig 1B). Cells were subjected to short-term (15–30 min), or long-term (2–6 hr), treatment with TNF. As shown in Fig 1C, short-term activation of NFκB was equivalent in EV and ST6-KD cells, whereas NFκB activation at 6 hrs following TNF treatment was blunted in ST6-KD cells. The loss of sustained NFκB activity in ST6-KD cells is in line with our prior data showing that knockdown of ST6Gal-I in U937 cells greatly increases TNF-induced apoptosis [22].

### Knockdown of ST6Gal-I expression diminishes long-term activation of NFκB by LPS

Considering the prominent role that NFκB plays in mediating inflammation, we evaluated a potential function for ST6Gal-I in regulating another pathway known to initiate NFκB activation, the LPS/TLR4 signaling axis. As with TNF stimulation of NFκB, ST6Gal-I activity had little effect on early (≤ 30 min) LPS-induced NFκB signaling (Fig 1D). However, ST6-KD cells treated with LPS for 6 hr demonstrated strikingly reduced phosphorylation of NFκB.

### ST6Gal-I activity modulates TNF and LPS-induced cytokine production

**Fig 2:**
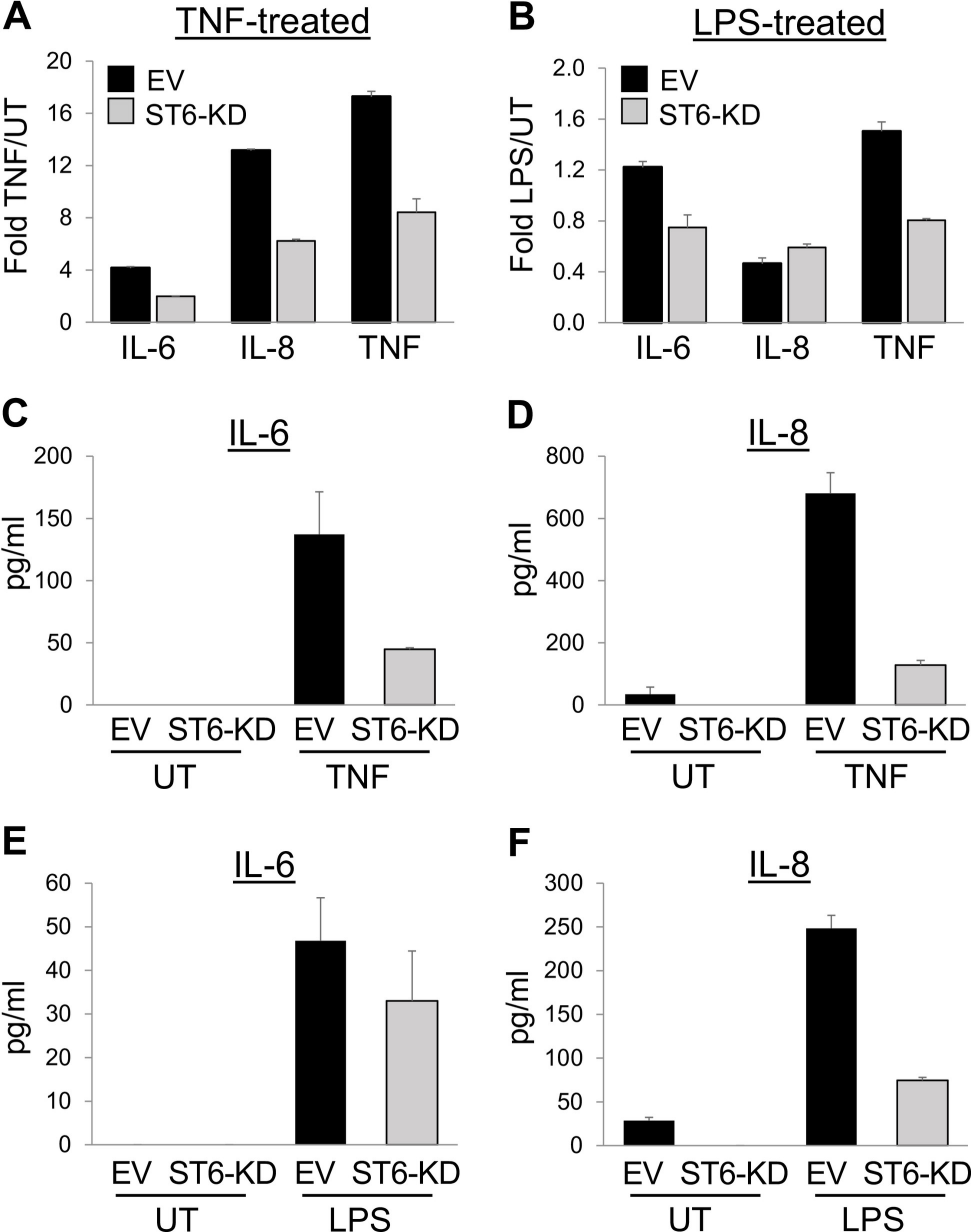
ST6Gal-I modulates TNF and LPS-induced cytokine production. (A-B) EV and ST6-KD cells were treated with TNF (A) or LPS (B) for 6 hrs, and induction of IL-6, IL-8 and TNF mRNA expression was measured by qRT-PCR. Values for TNF and LPS treated cells were normalized to their respective untreated controls. (C-D) EV and ST6-KD cells were treated with TNF for 6 hrs, or left untreated (UT), and ELISAs were conducted to measure secretion of IL-6 (C) and IL-8 (D). (E-F) Cells were treated with or without LPS for 6 hrs, and ELISAs used to measure secretion of IL-6 (E) and IL-8 (F). Graphs depict means and standard deviations from representative experiments. At least three independent experiments were conducted.

We evaluated the effects of α2–6 sialylation on known inflammatory targets of NFκB transcriptional activity, such as IL-6, IL-8 and TNF. qRT-PCR experiments revealed that ST6-KD cells had reduced mRNA expression of IL-6, IL-8 and TNF compared to EV cells following 6 hr of TNF treatment (Fig 2A). Likewise, LPS-treated ST6-KD cells had lower expression of IL-6 and TNF than EV cells, although no difference was noted in the expression of IL-8 (Fig 2B). To further assess cytokine production, ELISA assays were conducted on culture supernatants. Cells were either treated with TNF for 6 hrs, or left untreated (UT) for 6 hrs, and the conditioned media screened for levels of IL-6 and IL-8 (TNF secretion was not measured due to the presence of exogenous, recombinant TNF in the culture media). Relative to EV cells, ST6-KD cells secreted markedly lower levels of IL-6 and IL-8 in response to TNF (Fig 2C and 2D). Similarly, ELISAs were conducted on cells treated with or without LPS for 6 hr. As shown, EV cells secreted a greater amount of IL-6 and IL-8 than ST6-KD cells in response to LPS (Fig 2E and 2F). In contrast to IL-6 and IL-8, there was no detectable secretion of TNF from any of the cell populations, either untreated or LPS-treated (data not shown).

### ST6Gal-I activity promotes long-term LPS-induced STAT3 activation

**Fig 3:**
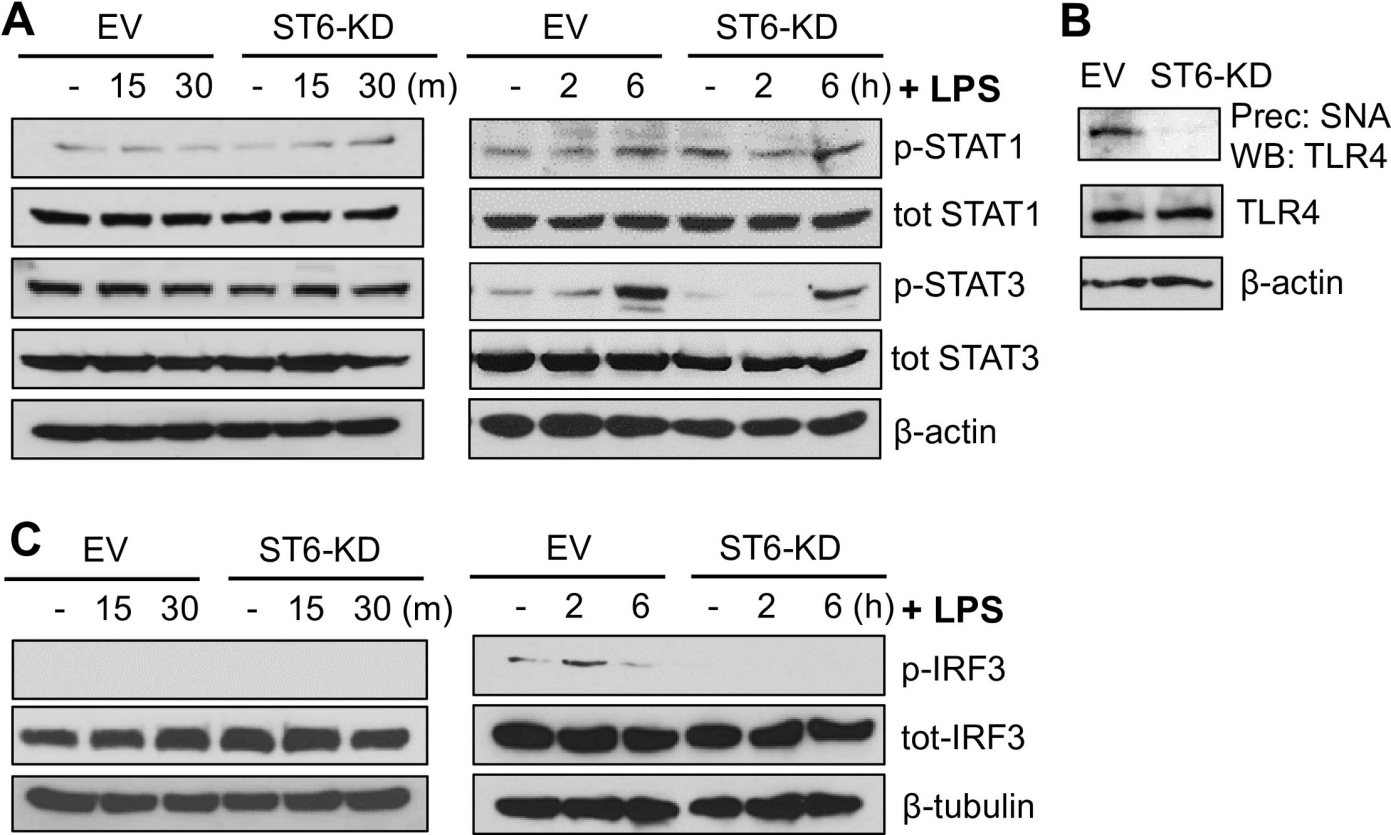
ST6Gal-I activity promotes long-term LPS-induced STAT3 activation. (A) U937 EV and ST6-KD cells were treated with LPS for short-term (15–30 min) or long-term (2–6 hr) timepoints, and lysates were immunoblotted for phosphorylated (activated) or total STAT1 and STAT3. (B) To assess whether TLR4 was α2–6 sialylated, lysates from EV and ST6-KD cells were incubated with agarose-conjugated SNA lectin and sialylated proteins were then precipitated by centrifugation. The precipitates were immunoblotted for TLR4 (upper blot). Total TLR4 expression was evaluated by immunoblotting for TLR4 in whole cell lysates (middle blot). (C) Lysates were probed for phosphorylated or total IRF3, a downstream mediator of LPS signaling. At least two independent experiments were performed for each immunoblot.

While the canonical LPS pathway centers on NFκB, it has been suggested that LPS can indirectly activate JAK/STAT signaling, further amplifying the inflammatory response to LPS [18, 19]. Accordingly, we evaluated the contribution of ST6Gal-I to LPS-induced JAK/STAT activation. Interestingly, we did not observe any rapid activation of either STAT1 or STAT3 in U937 cells upon LPS treatment (Fig 3A). Of note, U937 cells are thought to have some degree of constitutive STAT1 and STAT3 activation [27]. As with short-term LPS treatment, no differences in STAT1 activation were detected at 2 and 6 hr following incubation with LPS. However, LPS did activate STAT3 at long-term timepoints, and STAT3 phosphorylation was reduced in ST6-KD, compared to EV, cells at 2 and 6 hr after LPS treatment (Fig 3A).

### TLR4 is a substrate for ST6Gal-I mediated sialylation

Given that ST6Gal-I activity modulated LPS-induced NFκB and STAT3 activation, we evaluated whether the receptor responsible for propagating the LPS signal, TLR4, was a direct substrate for ST6Gal-I-mediated sialylation. Using agarose beads conjugated to SNA, α2–6 sialylated proteins were precipitated, and the precipitates were then immunoblotted for TLR4. As shown in Fig 3B, TLR4 was α2–6 sialylated in EV cells, however levels of α2–6 sialylated TLR4 were undetectable in ST6-KD cells. On the other hand, the total expression of TLR4 in whole cell lysates was unaffected by ST6Gal-I KD. Although the specific mechanisms by which TLR4 sialylation modulates downstream signaling remain to be examined, these results highlight a prospective molecule involved in the sialylation-driven modifications of LPS signaling.

### Knockdown of ST6Gal-I diminishes IRF3 activation

Because knockdown of ST6Gal-I led to a decrease in the long-term activation of NFκB by LPS, we evaluated activation of IRF3, a downstream target of the adaptor protein, TRIF. IRF3 is thought to be responsible for later-stages of TLR4-induced NFkB signaling [17]. While no detectable activation of IRF3 was noted at 15 or 30 min., IRF3 phosphorylation was observed at 2 hr., and levels were higher in the EV line at this time point (Fig 3C).

### ST6Gal-I activity has no effect on long-term JAK/STAT signaling induced by IFNγ, IL-6, and GM-CSF

**Fig 4:**
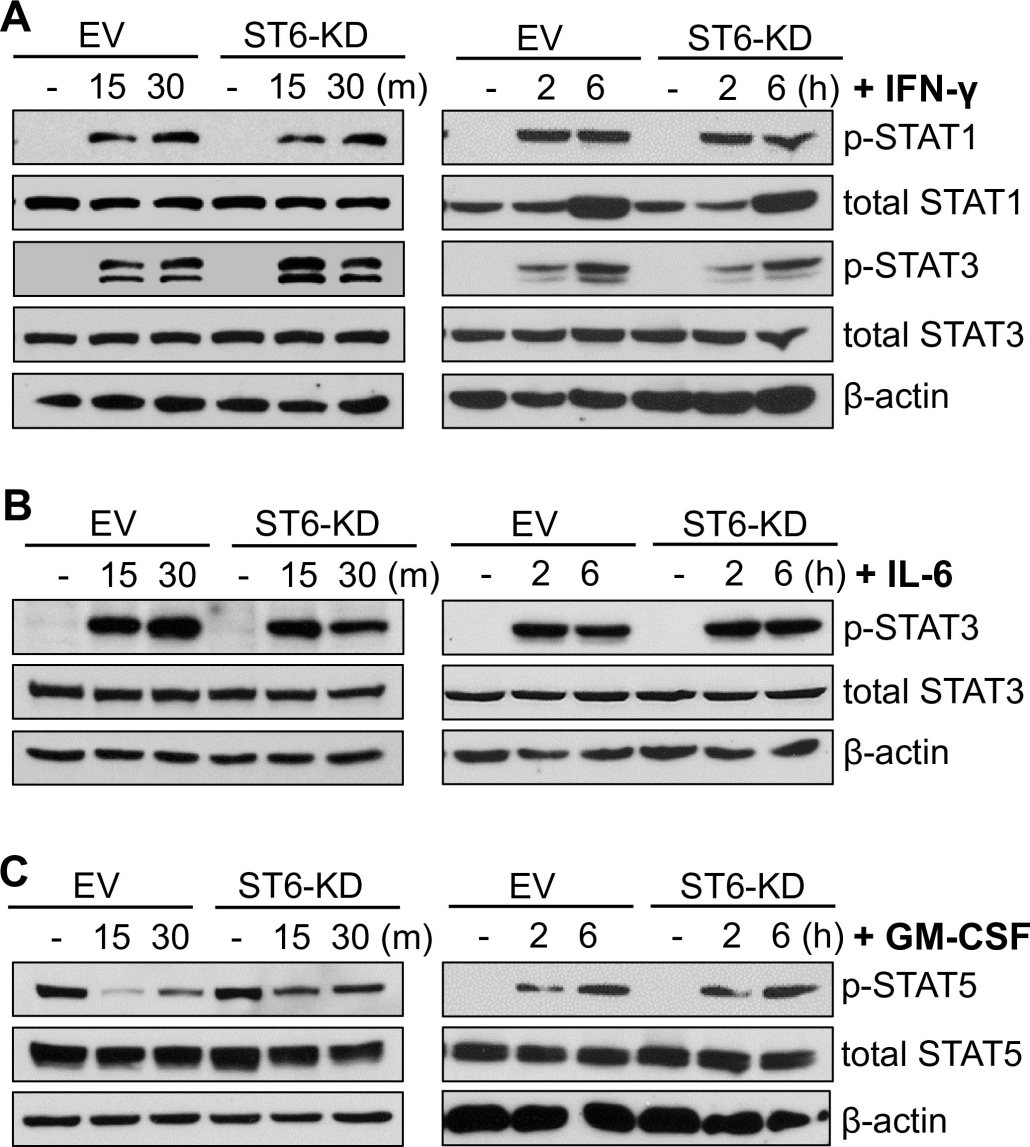
ST6Gal-I activity has a negligible effect on long-term JAK/STAT signaling induced by IFNγ, IL-6, or GM-CSF. (A) Cells treated with IFNγ for short-term or long-term timepoints were evaluated for activation of STAT1 and STAT3. (B) Cells treated with IL-6 for short-term or long-term timepoints were evaluated for activation of STAT3. (C) Cells treated with GM-CSF for short-term or long-term timepoints were evaluated for activation of STAT5. Immunoblots are representative of at least two independent experiments.

Since ST6Gal-I activity influenced TNF and LPS-induced signaling, we interrogated a role for ST6Gal-I in other cytokine-mediated inflammatory pathways. U937 cells were treated with IFNγ and examined for activation of STAT1 and STAT3, both of which are well-known downstream targets of IFNγ. As shown in Fig 4A, ST6Gal-I KD had little effect on the IFNγ-dependent activation of STAT1 or STAT3 at any of the timepoints measured. We next evaluated STAT3 activation by IL-6. At 15 min following IL-6 treatment, comparable amounts of p-STAT3 were detected in EV and ST6-KD cells, although at 30 min, there appeared to be a modest decrease in p-STAT3 levels in ST6-KD cells (Fig 4B). At 2 and 6 hr after IL-6 treatment, STAT3 activation was equivalent in EV and ST6-KD cells (Fig 4B). Finally, we monitored the effects of GM-CSF on STAT5 activation. At 15 and 30 min. following GM-CSF treatment, pSTAT5 levels were lower than in untreated cells, suggesting an initial inhibitory effect of GM-CSF (Fig 4C). However, GM-CSF-treated ST6-KD cells appeared to retain higher levels of p-STAT5 than EV cells at these early time points. In contrast, STAT5 was strongly activated by GM-CSF at 2 and 6 hrs after treatment, although the levels of p-STAT5 were comparable in EV and ST6-KD cells (Fig 4C). In the aggregate, these results suggest that, although some relatively minor differences were observed in short-term STAT signaling, ST6Gal-I activity had no major effect on sustained signaling by IFNγ, IL-6 or GM-CSF, in marked contrast to signaling induced by TNF and LPS. These data suggest that ST6Gal-I-mediated sialylation regulates the activity of specific receptors and their cognate signaling pathways, rather than fundamentally altering global inflammatory signaling.

### Deletion of *St6gal1* in primary macrophages suppresses TNF and LPS signaling

**Fig 5:**
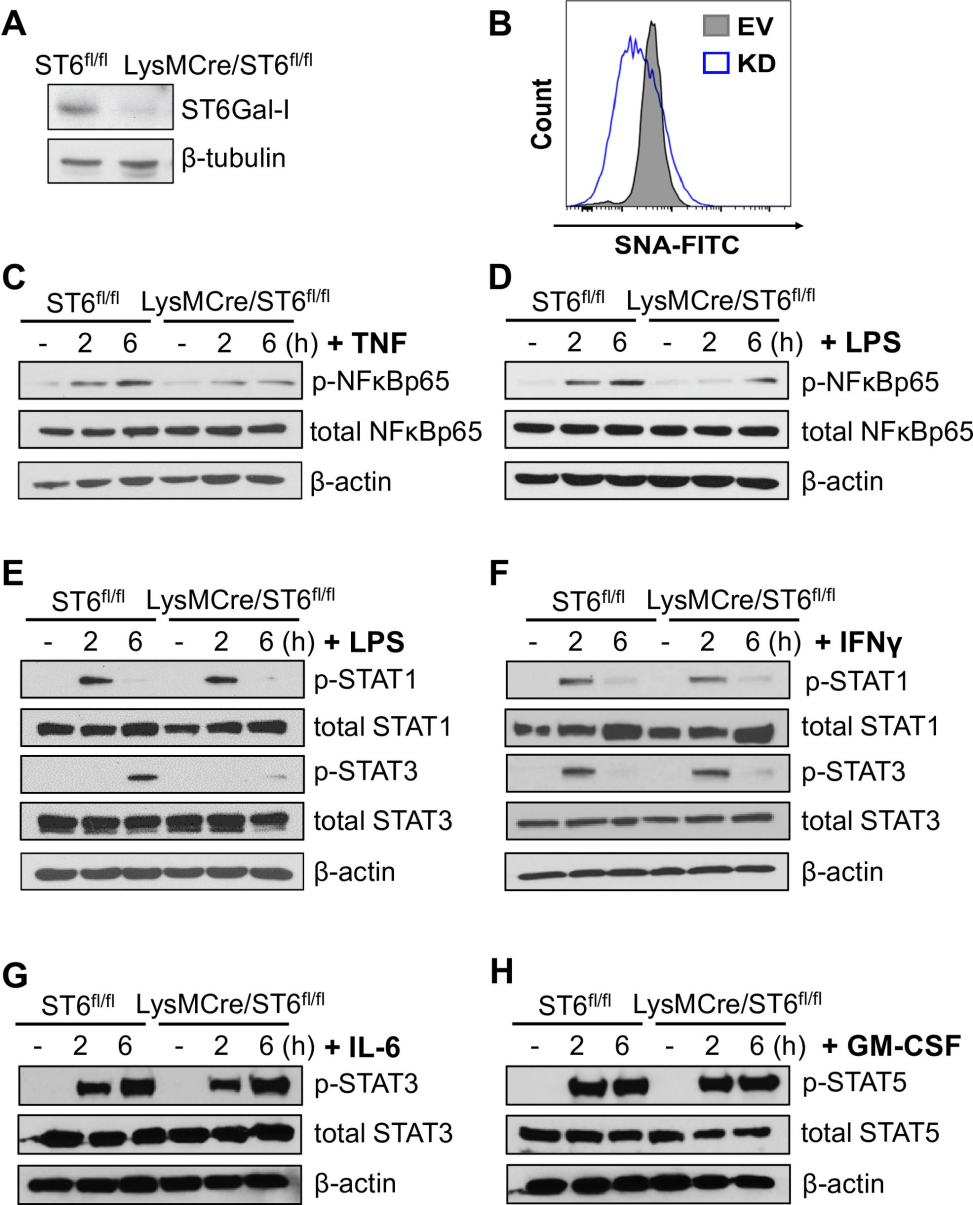
Deletion of *St6gal1* in primary macrophages suppresses TNF and LPS signaling. (A) Mice with floxed *St6gal1* were cross-bred with mice expressing Cre recombinase under control of the LysM promoter (LysM/Cre-ST6^fl/fl^). Mice with floxed *St6gal1*, but lacking Cre recombinase (ST6 ^fl/fl^), served as the control population. Monocytes were isolated from the bone marrow and exposed to M-CSF for 4–6 days to obtain BMDMs. Knockout of *St6gal1* in LysMCre/ST6^fl/fl^ mice was confirmed by immunoblotting. (B) Control and *St6gal1* knockout BMDMs were stained with SNA-FITC and surface sialylation analyzed with flow cytometry. (C) Control and *St6gal1* knockout BMDMs were treated with TNF for 2 or 6 hr, and lysates immunoblotted for phospho- and total NFκBp65. (D) Control and *St6gal1* knockout BMDMs were treated with LPS for 2 or 6 hr, and lysates immunoblotted for phospho- and total NFκBp65. (E) Control and *St6gal1* knockout BMDMs were treated with LPS for 2 or 6 hr, and lysates immunoblotted for phospho- and total STAT1 and STAT3. (F) Control and *St6gal1* knockout BMDMs were treated with IFNγ for 2 or 6 hr, and lysates immunoblotted for phospho- and total STAT1 and STAT3. (G) Control and *St6gal1* knockout BMDMs were treated with IL-6 for 2 or 6 hr, and lysates immunoblotted for phospho- and total STAT3. (H) Control and *St6gal1* knockout BMDMs were treated with GM-CSF for 2 or 6 hr, and lysates were immunoblotted for phospho- and total STAT5.

To corroborate the findings gleaned from U937 monocytic cells, we generated mice lacking ST6Gal-I expression in myeloid lineage cells. C57BL/6 mice expressing a floxed *St6gal1* gene were crossed to LysM-Cre mice (“LysMCre/ST6^fl/fl^”). Mice with floxed *St6gal1*, but lacking Cre-recombinase expression (“ST6^fl/fl^”), were used as the control. Monocytes were isolated from the bone marrow of the mice and cultured under established conditions to generate BMDMs. Cre-mediated deletion of *St6gal1* in LysMCre/ST6^fl/fl^ mice was confirmed by immunoblotting for ST6Gal-I in BMDM lysates (Fig 5A), and SNA staining showed that BMDMs lacking ST6Gal-I had reduced α2–6 sialylation (Fig 5B). We then examined signaling events occurring 2 to 6 hr following cytokine treatment. Similar to U937 ST6Gal-I KD cells, the long-term activation of NFκB by TNF was dampened in BMDMs from *St6gal1* knock-out mice (Fig 5C). Interestingly, we did not observe any appreciable apoptosis in TNF-treated BMDMs (not shown). LPS-dependent activation of NFκB was also reduced in *St6gal1* knockout BMDMs as compared with wild-type cells (Fig 5D). Additionally, *St6gal1* knockout mice had decreased activation of STAT3, whereas STAT1 activation was unaffected (Fig 5E). We then evaluated STAT signaling downstream of IFNγ, IL-6, and GM-CSF (Fig 5F–5H, respectively), and found that activation of the relevant STATs by these three cytokines was equivalent in control and *St6gal1* knock-out BMDMs. Taken together, the data generated from U937 cells and BMDMs suggest that ST6Gal-I activity selectively promotes the sustained activation of three signaling nodes, TNF/NFkB, LPS/NFkB and LPS/STAT3.

## Discussion

TNF is a potent pro-inflammatory cytokine released in response to trauma or infection, and is among the most abundant early mediators in inflamed tissues [28]. TNF expression is also induced by bacterial-derived LPS, which signals though pattern-recognition receptors (PRRs) such as TLR4 [17]. TNF and LPS play critical roles in macrophage activation, regulating the production of numerous inflammatory cytokines. Additionally, TNF can promote macrophage survival or differentiation, depending upon context [28]. NFκB is one of the principal downstream signaling mediators for both LPS and TNF. The regulation of NFκB signaling is complex due to the presence of numerous intracellular activators and inhibitors. The temporal kinetics of NFκB activation play a large part in functional outcomes. In some cell types, for example, fibroblasts, NFκB signaling is oscillatory, with NFκB activation rapidly turning on and off [29]. However, macrophages typically respond with a single NFκB nuclear translocation event, which persists for as long as the stimulus remains [29]. Other investigators have reported that the long-lasting persistence of nuclear-localized (active) NFκB in macrophages is correlated within enriched expression of inflammatory cytokine genes [30].

In the current study, we find that ST6Gal-I activity prolongs NFkB activation in response to TNF and LPS, which we hypothesize would promote the extended secretion of cytokines within the tissue microenvironment. Using monocytic cell lines engineered with ST6Gal-I knockdown or BMDMs with ST6Gal-I knockout, we determined that cells lacking ST6Gal-I had diminished long-term activation of NFκB, and reduced cytokine production. Furthermore, LPS-induced activation of IRF3 and STAT3 was attenuated in ST6Gal-I deficient cells. To assess the specificity of ST6Gal-I sialylation in modulating inflammatory signaling, we monitored the activation of the JAK/STAT pathway by other cytokines. Interestingly, loss of ST6Gal-I had no apparent effect on either short- or long-term activation of STAT1 or STAT3 by IFNγ. In response to treatment with IL-6 or GM-CSF, minor differences were noted in the rapid activation of STATs, however long-term signaling induced by IL-6 or GM-CSF was comparable in cells with high or low ST6Gal-I expression. These findings suggest that TNF and LPS are predominant pathways by which ST6Gal-I influences inflammatory signaling.

ST6Gal-I’s role in TNF signaling is mediated, at least in part, through an α2–6 sialylation-dependent block in TNFR1 internalization [21]. However, the mechanism by which α2–6 sialylation regulates TLR4 signaling remains undetermined. In addition to modulating surface retention of receptors, α2–6 sialylation has the potential to modify receptor conformation and clustering, and could also impact ligand binding. Importantly, dimerization of TLR4 is essential for its activation [17], and TLR4 internalization can impact signaling through recruitment of TRIF instead of MyD88, activating both NFκB and IRF3 [31–33]. ST6Gal-I-mediated sialylation is known to affect the oligomerization and/or internalization of several surface receptors including CD45 and PECAM1 [34, 35]. Adding to the possible mechanisms of TLR4 regulation by ST6Gal-I, *N*-linked glycosylation of the TLR4-associated molecules, CD14 and MD-2, is essential for their function and stability [36, 37].

The relationship between sialylation and the LPS/TLR4 pathway has been investigated by others. As in our work, the SNA lectin was used to determine that TLR4 is α2–6 sialylated [26]. The functional effects of TLR4 sialylation were also examined, however in this case, receptor sialylation was manipulated by treating the cell surface with sialidase enzymes rather than through direct modulation of ST6Gal-I expression. Following sialidase treatment, it was shown that removal of surface sialic acids increased LPS-induced NFκB signaling [26, 38]. However, the sialidase used in these prior studies cleaves both α2–3 and α2–6 sialic acids, which may not be biologically equivalent to selective changes in α2–6 sialylation induced by alterations in ST6Gal-I expression. Fluctuations in ST6Gal-I levels are biologically relevant, in that ST6Gal-I expression is dynamically regulated in certain immune cell populations [3, 39, 40], and typically upregulated in epithelial malignancies [41, 42]. Moreover, another group utilized a sialidase specific for α2–3 sialic acids, along with lectins that block either α2–3 or α2–6 sialic acids, and determined that it was the α2–3, but not α2–6, sialic acids that hindered TLR4 receptor activation and signaling [43]. In contrast to this work, our studies suggest that α2–6 sialylation of TLR4 enhances LPS-dependent signaling.

The combined results presented in this manuscript add to the accumulating evidence implicating ST6Gal-I activity in immune cell signaling and function. Murine knockout of *St6gal1* impairs B cell maturation and antibody production [4] as well as thymopoiesis [3]. Additionally, deletion of *St6gal1* enhances the neutrophilic and Th2 response in murine models of peritonitis and allergic pulmonary inflammation, respectively [2, 44]. As well, extracellular sialylation of hematopoietic stem cells by secreted ST6Gal-I depletes neutrophil reserves, and upon challenge with LPS, results in decreased neutrophil infiltration [1]. In other studies, a highly pathogenic subset of stem-like CD4 T cells was identified in a mouse model of colitis. Significantly, these T cells have enriched ST6Gal-I and are also resistant to apoptosis [39]. Many of these aforementioned studies indicate that ST6Gal-I is vital for immune cell differentiation and/or activation, suggesting that ST6Gal-I regulates immune cell fate. Notably, ST6Gal-I levels are high in some immature or naive immune cell populations, including dendritic cells and T cells, but decline upon activation and/or differentiation of these cells [45–48].

In conclusion, the current findings augment our fundamental understanding of how ST6Gal-I-directed sialylation regulates two signaling pathways that are highly involved in inflammation, NFκB and JAK/STAT. They also supplement the breadth of literature implicating ST6Gal-I as a major mediator of immunity. Ultimately, these results help bridge the knowledge gap between the cellular effects of receptor sialylation and the immune outcomes driven by ST6Gal-I.

## Supporting information

S1 Fig (PDF)
