# Supplementary material for: Regulation of inflammatory signaling by the ST6Gal-I sialyltransferase
Source: PLoS One. 2020 Nov 9;15(11):e0241850. doi: 10.1371/journal.pone.0241850 (PMC7652342; doi:10.1371/journal.pone.0241850)
Supplement: S1 Fig — (PDF) [file pone.0241850.s001.pdf]

Figure 1A-B

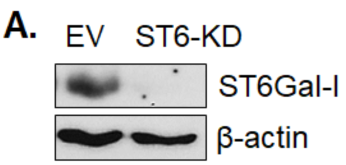
A.
EV
ST6-KD
ST6Gal-I
β-actin

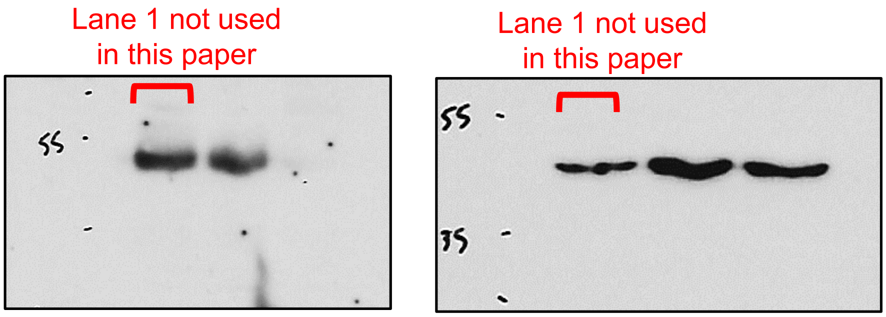
Lane 1 not used in this paper
55
Lane 1 not used in this paper
55
35

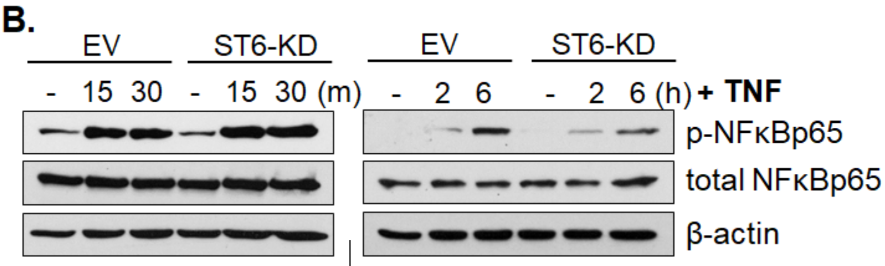
B.
EV
ST6-KD
EV
ST6-KD
- 15 30 - 15 30 (m)
- 2 6 - 2 6 (h)
+ TNF
p-NFκBp65
total NFκBp65
β-actin

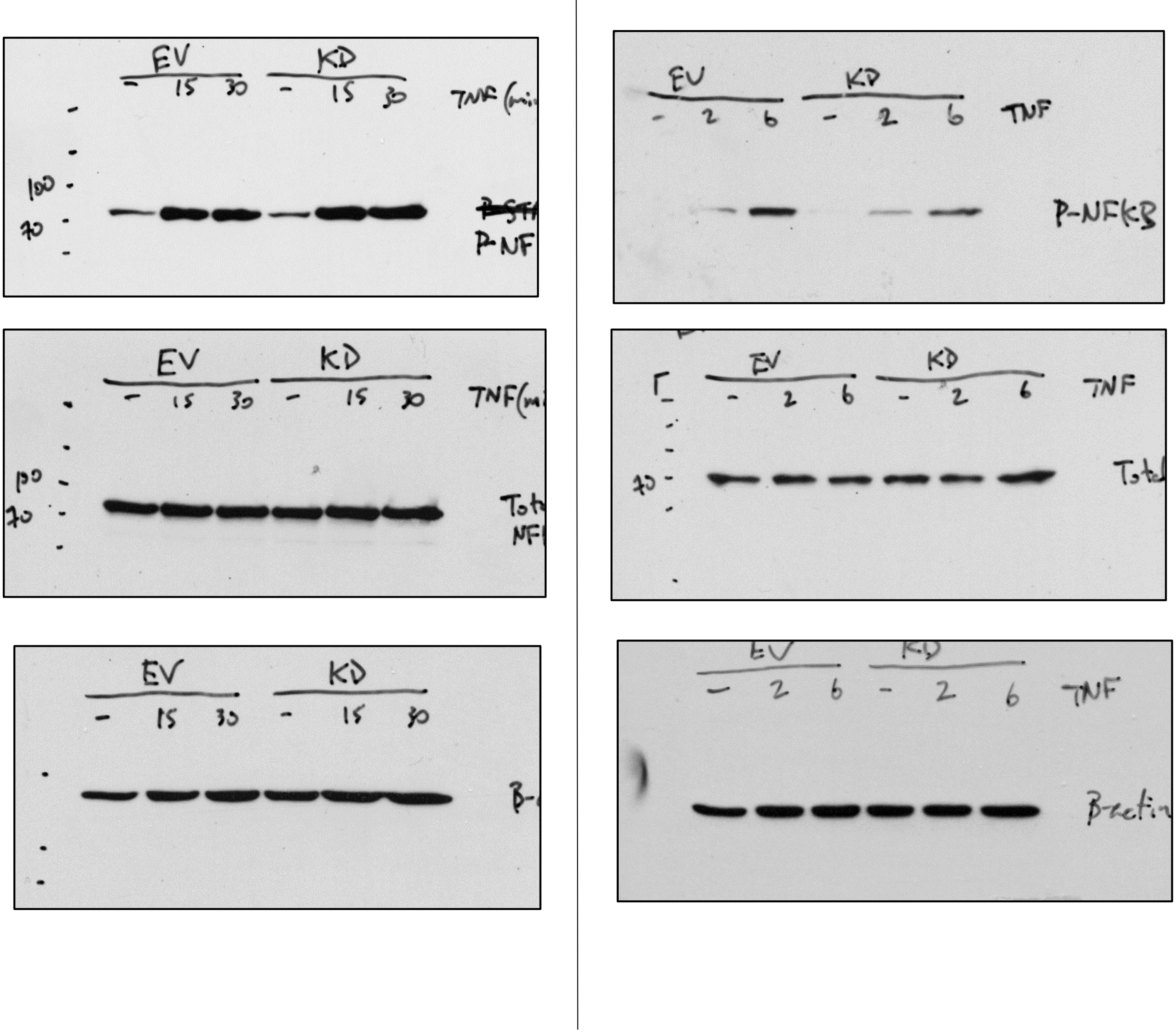
EV
KD
TNF
P-NFKB
β-actin

Figure 1C

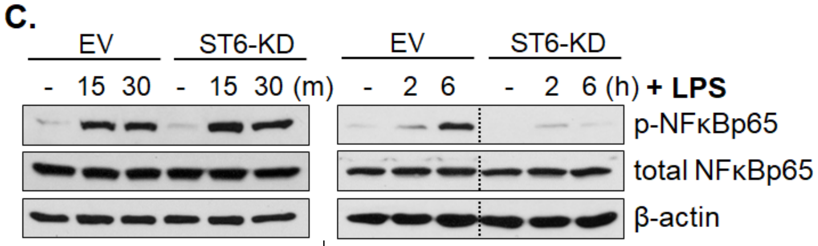

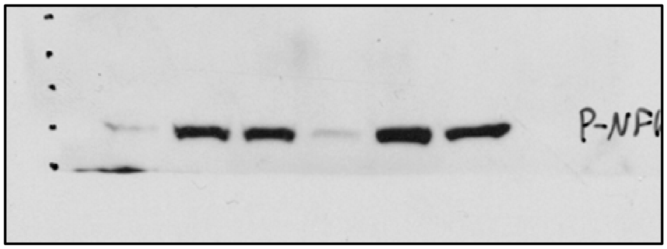

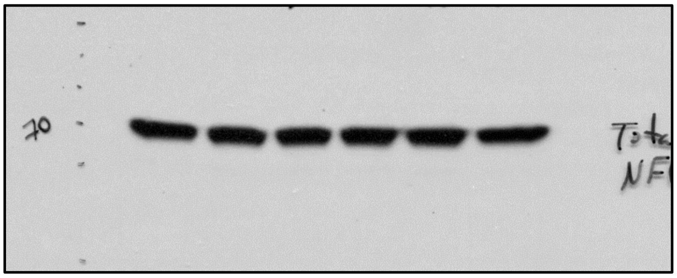

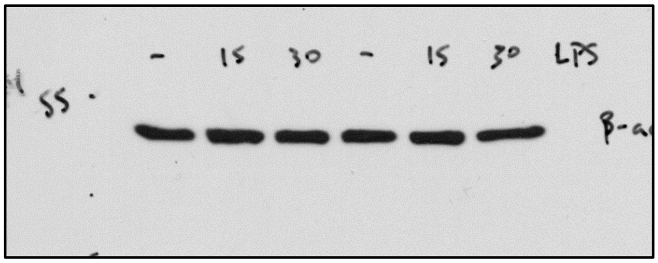

Lanes 4-6 not used in this paper

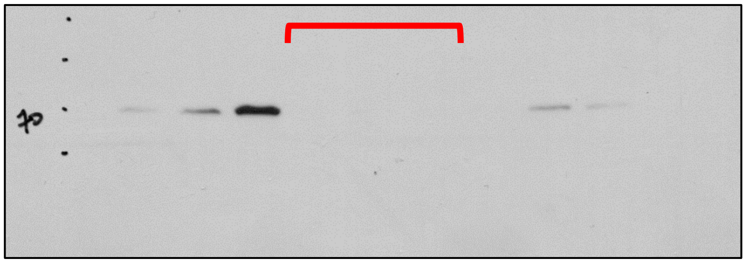

Lanes 4-6 not used in this paper

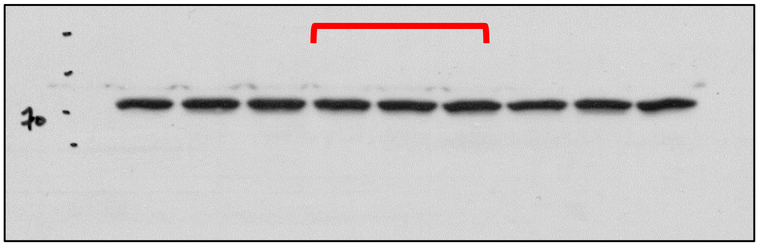

Lanes 4-6 not used in this paper

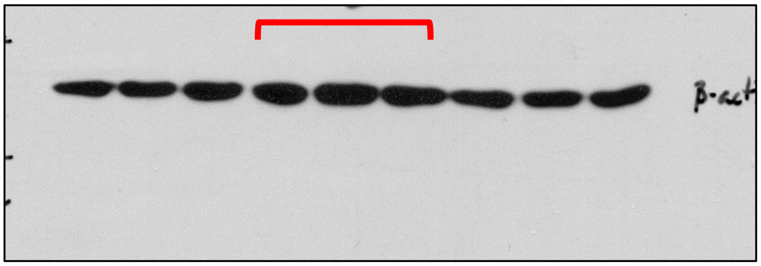

Figure 2

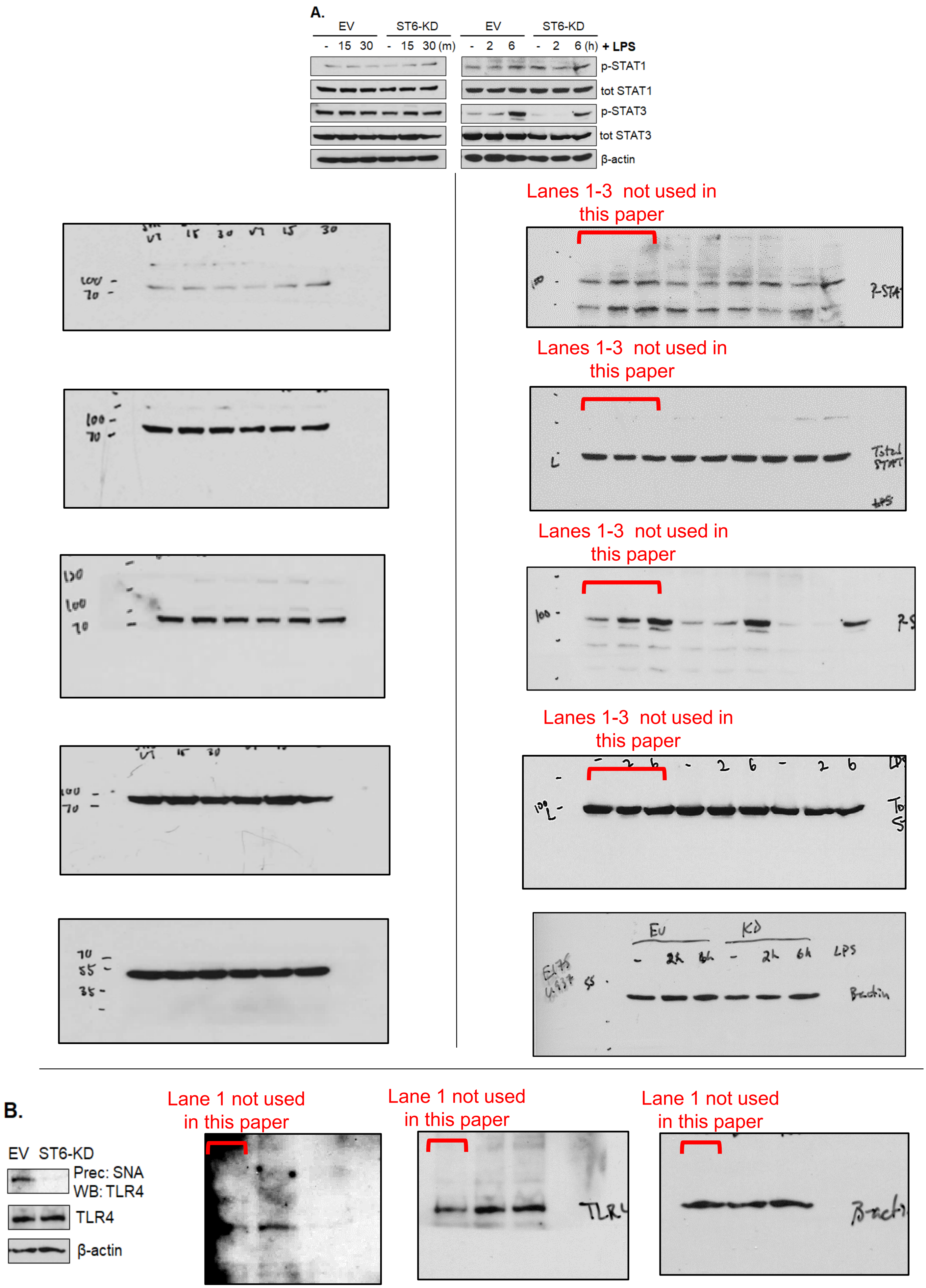
A.
EV
ST6-KD
EV
ST6-KD
- 15 30 - 15 30 (m) - 2 6 - 2 6 (h) + LPS
p-STAT1
tot STAT1
p-STAT3
tot STAT3
β-actin
Lanes 1-3 not used in this paper
Lanes 1-3 not used in this paper
Lanes 1-3 not used in this paper
Lanes 1-3 not used in this paper
B.
EV ST6-KD
Prec: SNA
WB: TLR4
TLR4
β-actin
Lane 1 not used in this paper
Lane 1 not used in this paper
Lane 1 not used in this paper

Figure 3 A

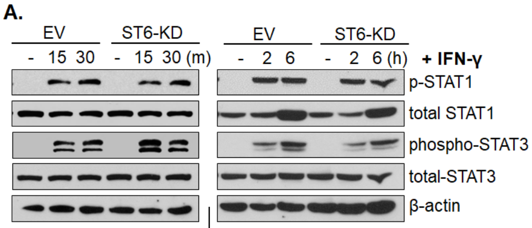

Lanes 7-9 not used in this paper

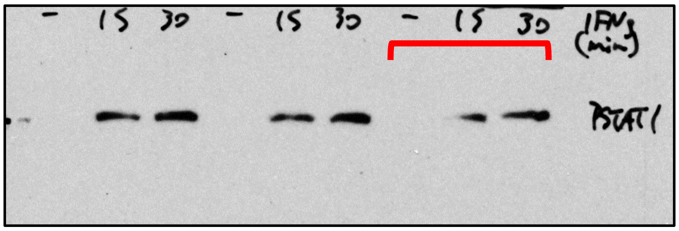

Lanes 1-3 not used in this paper

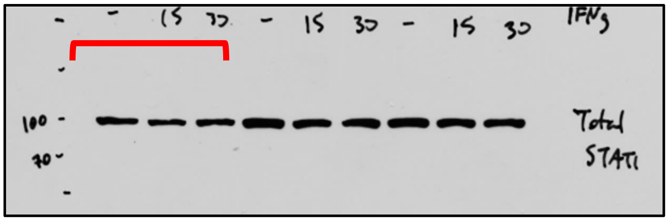

Lanes 7-9 not used in this paper

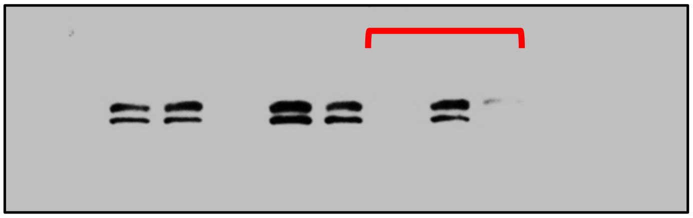

Lanes 1-3 not used in this paper

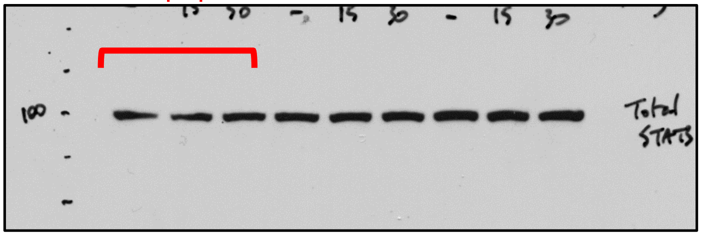

Lanes 1-3 not used in this paper

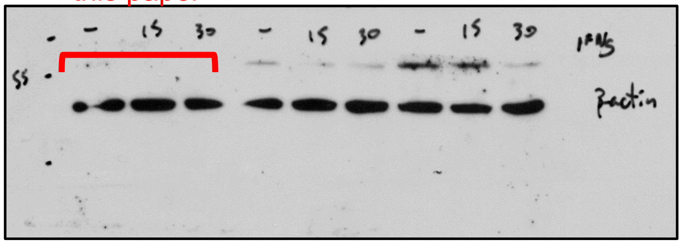

Lanes 1-3 not used in this paper

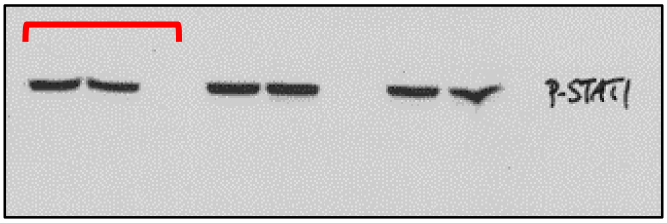

Lanes 1-3 not used in this paper

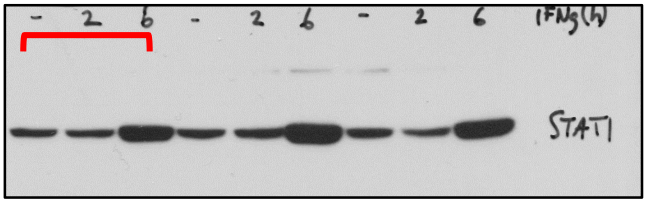

Lanes 1-3 not used in this paper

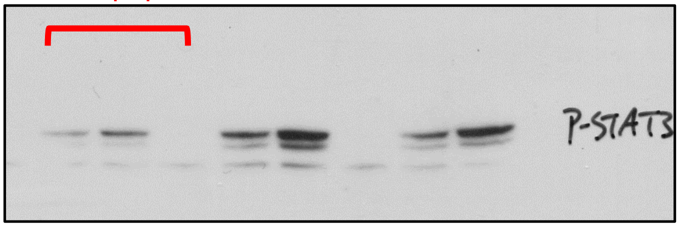

Lanes 1-3 not used in this paper

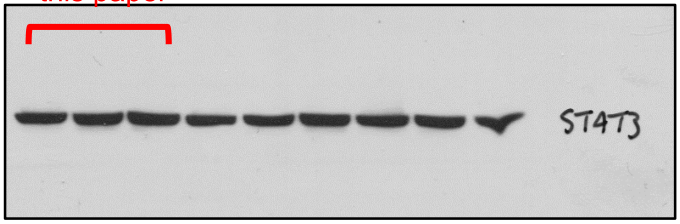

Lanes 1-3 not used in this paper

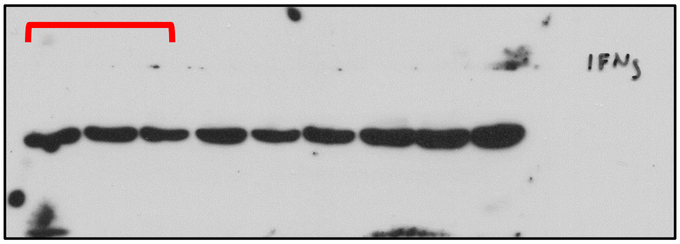

Figure 3 B & C

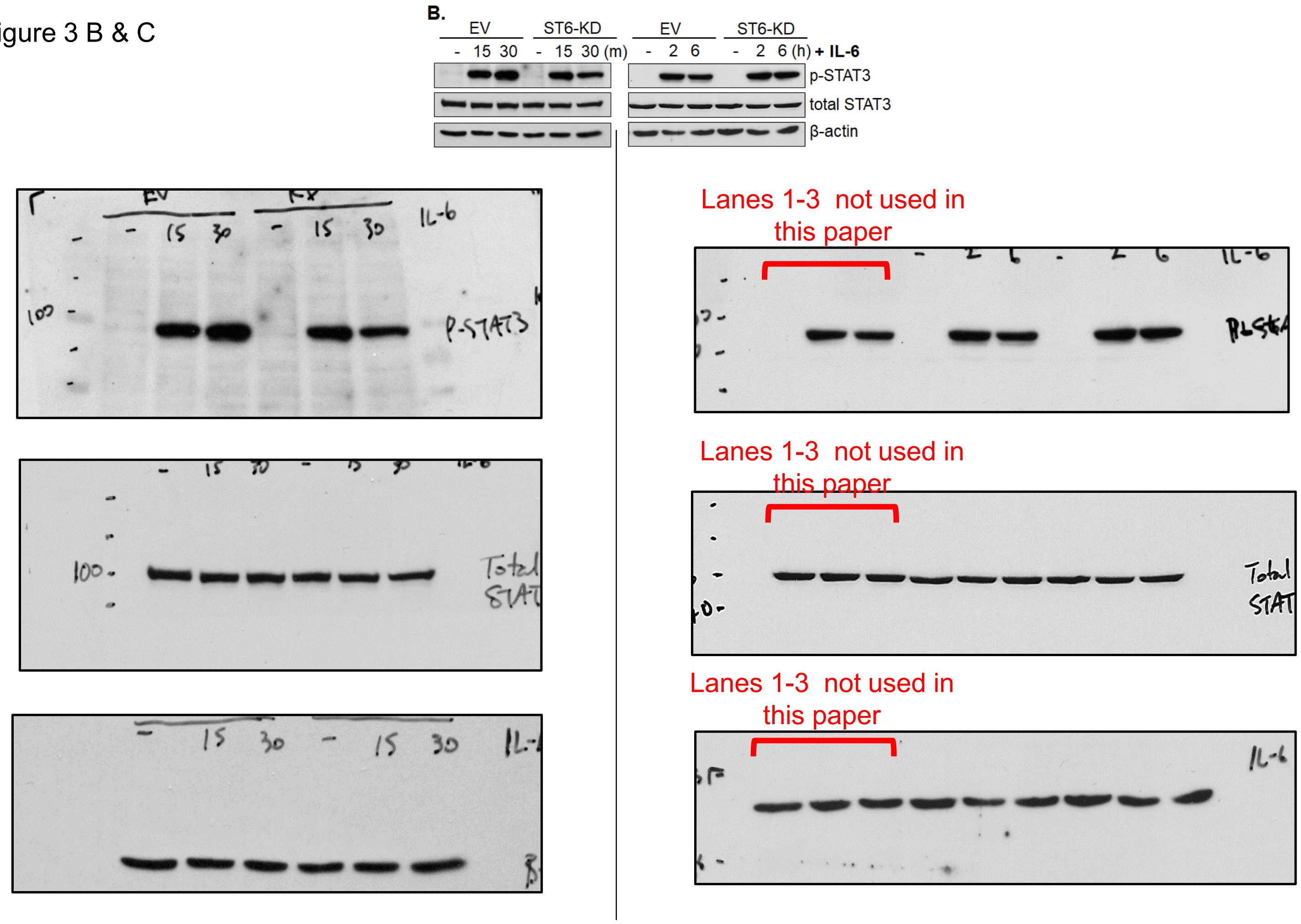
B.
EV
ST6-KD
- 15 30
- 15 30 (m)
EV
ST6-KD
- 2 6
- 2 6 (h) + IL-6
p-STAT3
total STAT3
β-actin
Lanes 1-3 not used in this paper
Lanes 1-3 not used in this paper
Lanes 1-3 not used in this paper

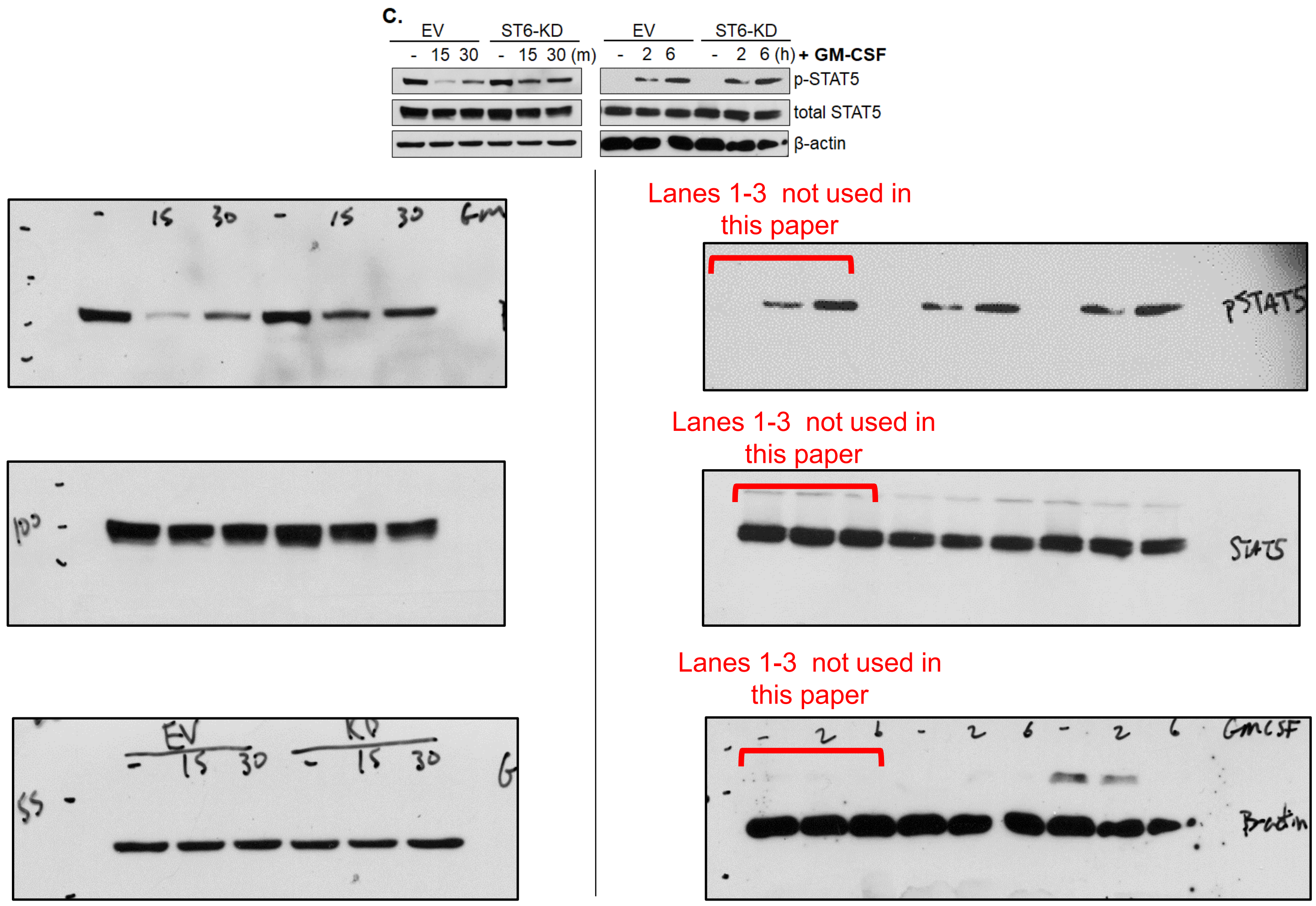
C.
EV
ST6-KD
- 15 30
- 15 30 (m)
EV
ST6-KD
- 2 6
- 2 6 (h) + GM-CSF
p-STAT5
total STAT5
β-actin
Lanes 1-3 not used in this paper
Lanes 1-3 not used in this paper
Lanes 1-3 not used in this paper

Figure 4 A-C

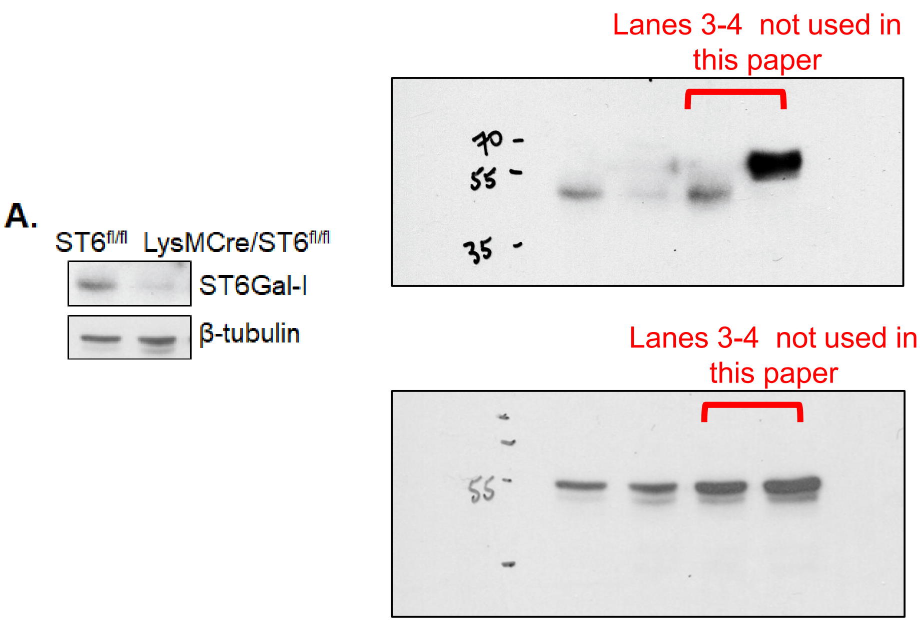
Lanes 3-4 not used in this paper
A.
ST6fl/fl LysMCre/ST6fl/fl
ST6Gal-I
β-tubulin
70
55
35
Lanes 3-4 not used in this paper
55

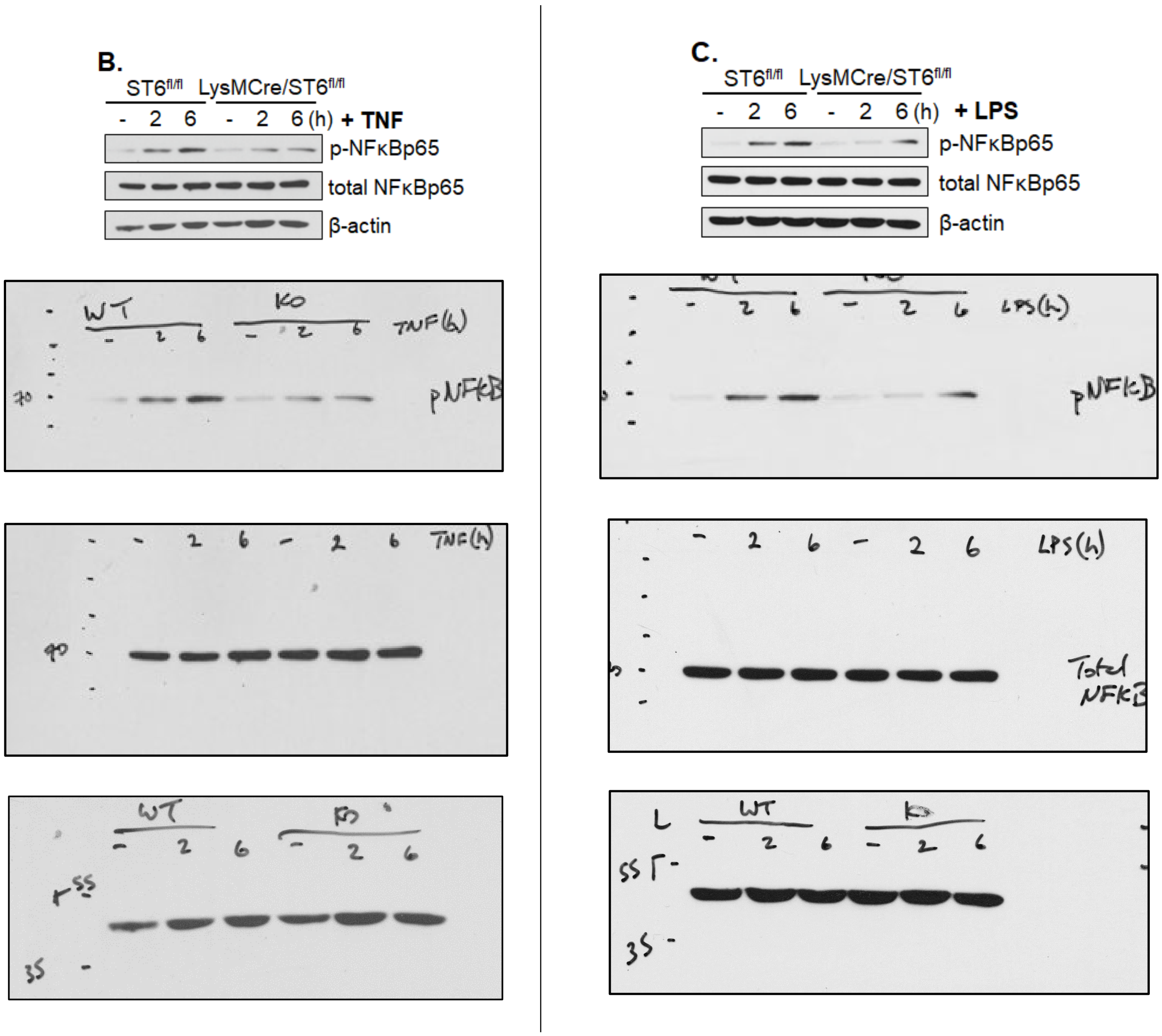
B.
ST6fl/fl LysMCre/ST6fl/fl
- 2 6 - 2 6 (h) + TNF
p-NFκBp65
total NFκBp65
β-actin
WT
KO
- 2 6 - 2 6 TNF(h)
pNFkB
70
- 2 6 - 2 6 TNF(h)
70
WT
KO
- 2 6 - 2 6
55
35
C.
ST6fl/fl LysMCre/ST6fl/fl
- 2 6 - 2 6 (h) + LPS
p-NFκBp65
total NFκBp65
β-actin
- 2 6 - 2 6 LPS(h)
pNFkB
- 2 6 - 2 6 LPS(h)
Total NFkB
WT
KO
- 2 6 - 2 6
55
35

Figure 4 D-E

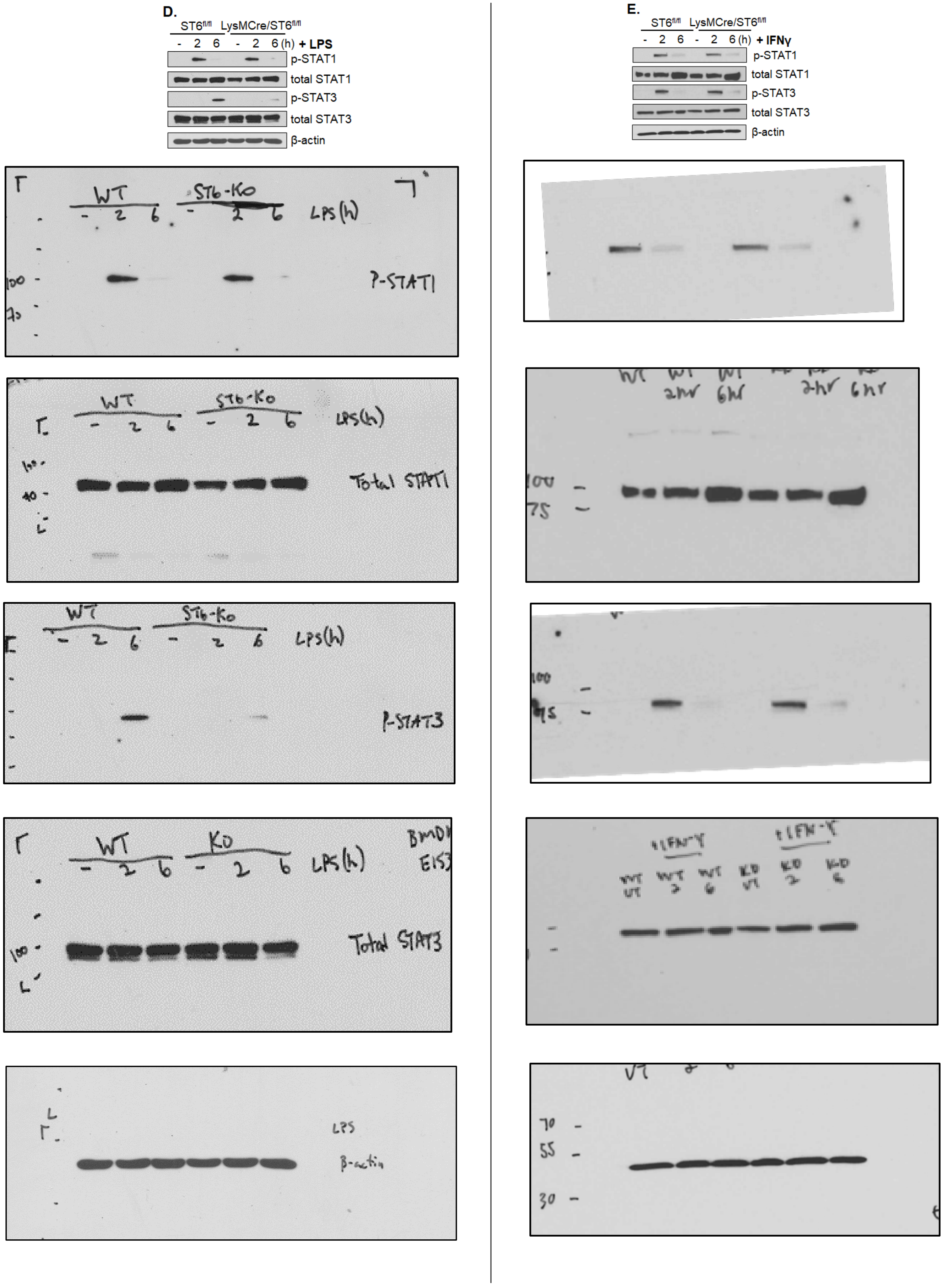
D.
ST6fl/fl LysMCre/ST6fl/fl
- 2 6 - 2 6 (h) + LPS
p-STAT1
total STAT1
p-STAT3
total STAT3
β-actin
E.
ST6fl/fl LysMCre/ST6fl/fl
- 2 6 - 2 6 (h) + IFNγ
p-STAT1
total STAT1
p-STAT3
total STAT3
β-actin

Figure 4 F-G

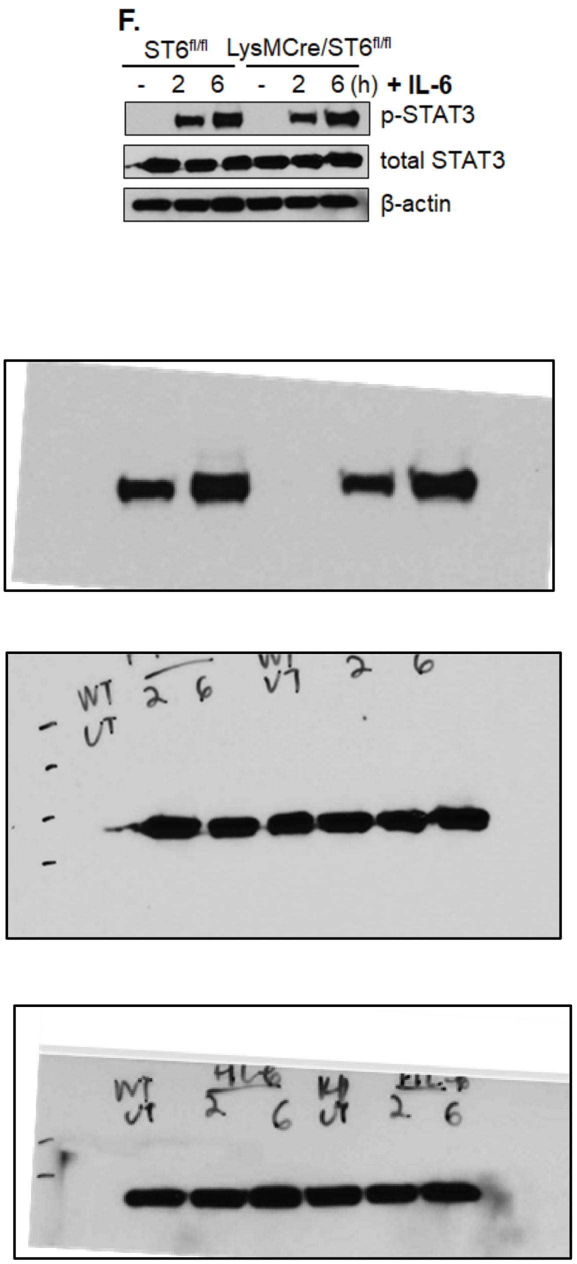
F.
ST6fl/fl
LysMCre/ST6fl/fl
- 2 6 - 2 6 (h) + IL-6
p-STAT3
total STAT3
β-actin

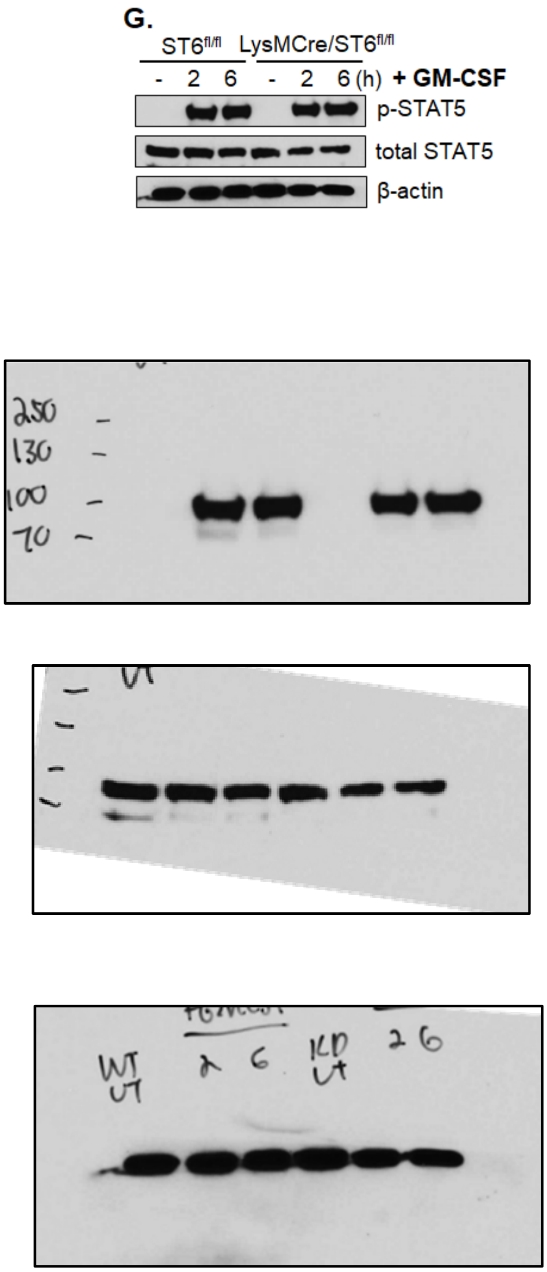
G.
ST6fl/fl
LysMCre/ST6fl/fl
- 2 6 - 2 6 (h) + GM-CSF
p-STAT5
total STAT5
β-actin
250
130
100
70
